# Estimating Left Ventricular Mass from the Electrocardiogram across the Spectrum of LV Mass from Normal to Increased LV Mass in an Older Age Group

**DOI:** 10.1155/2024/6634222

**Published:** 2024-03-11

**Authors:** Simon W. Rabkin, Jeremy C. J. Zhou

**Affiliations:** ^1^University of British Columbia, Vancouver, B.C., Canada; ^2^Division of Cardiology, Vancouver, B.C., Canada

## Abstract

**Objectives:**

To examine the relationship of QRS voltages and left ventricular (LV) mass across the spectrum of individuals with different LV mass.

**Methods:**

Twenty QRS voltage measurements or combinations were determined in a consecutive series of 159 adults with an ECG and echocardiogram without previous myocardial infarction, left or right bundle branch block, pre-excitation, or electronic pacemaker.

**Results:**

The four strongest and significant correlations between QRS and LV mass were S in V4, deepest S wave in any precordial lead plus S in V4, S in V3, and S in V3 plus R in AVL times QRS duration. For men, the strength of the relationships were S in V3 (*F* = 33.8), deepest S wave in any precordial lead plus S V4 (*F* = 33.7), S in V3 plus R aVL (*F* = 29.9), S in V4 (*F* = 29.79), and deepest S in precordial leads (*F* = 17.9). The R wave in AVL alone did not correlate with LV mass. Criteria using the R wave in lateral precordial leads did not correlate as strongly with LV mass. For women, only S in V4 significantly correlated with LV mass. Overall, the R wave voltage in limb leads (AVL I or II) did not correlate with precordial S wave amplitudes. Univariate and multivariate analysis showed that some but not all QRS voltages correlated with each other. In multivariate analysis, using only single variables and not combination of QRS variables, the only significant relationship between QRS voltage and left ventricular mass was for men the S in V3 (*p* = 0.04) and for women S in V4 (*p* = 0.016) and R in V6 (*p* = 0.04).

**Conclusion:**

The S wave in V3 and V4 correlate most strongly with LV mass while the R wave in limb leads, including AVL, do not correlate.

## 1. Introduction

The QRS voltage in the 12-lead ECG has been recognized as a significant predictor of subsequent cardiovascular events in various populations [1–9]. The fundamental basis of this risk factor for subsequent cardiovascular morbidity and mortality is usually attributed to the QRS voltage as a reflection of left ventricular mass or left ventricular hypertrophy (LVH), albeit not a highly sensitive indicator of LVH [10–17]. Increased left ventricular mass has deleterious consequences for adequate myocardial perfusion and is a substrate for serious cardiac arrhythmias [18–21] which may explain the relationship between ECG-LVH and subsequent cardiovascular events [1–9].

Studies have mainly focused on developing ECG criteria for the diagnosis of left ventricular hypertrophy and defining the sensitivity and specificity of each criterion. In contrast, there are very little data available on the QRS voltage in the absence of left ventricular hypertrophy or across the spectrum of individuals with different amounts of left ventricular mass. Verdecchia et al. studied untreated patients with hypertension without LVH on their ECG and concluded that QRS voltage was a predictor of an adverse outcome and the relationship of the ECG to left ventricular mass was mainly evident for R voltages in the limb leads (AVL, I, and III) [22]. In contrast, recent studies have concluded that LV mass is closely related to the S wave voltage in precordial leads including a relatively new criterion for LVH [15]. Underscoring the importance of the QRS on the 12-lead ECG is the recommendation for measurement of the 12-lead ECG as part of the basic assessment of patients for or with cardiovascular disease [23, 24]. The objective of this study was to examine the relationship of QRS voltage to left ventricular mass across the spectrum of patients with cardiovascular disease and left ventricular mass.

## 2. Methods

### 2.1. Subjects

A consecutive series of patients were retrospectively obtained from adults attending an outpatient Cardiology Clinic who had an echocardiogram. The entry criteria were age over 19 years and an echocardiogram and electrocardiogram within a two month time frame. The exclusion criteria were previous myocardial infarction, left or right bundle branch block, pre-excitation syndrome, or electronic pacemaker. The study was approved by our Institution's Committee on Research, and informed consent had been previously obtained from patients to use their examination data.

### 2.2. Measurements

Individuals had undergone a standard 12-lead ECG. All 12-lead electrocardiograms were recorded digitally on Muse™, version 9.0 SP6 (General Electric, Boston, USA). A total of 159 patients with echocardiogram and ECG data were included. ECGs were analyzed in detail. The amplitudes of the R waves of leads aVL, I, III, V4, V5, and V6, and the amplitudes of the S waves of leads V1, V2, V3, and V4 were measured. In addition, the deepest S wave in any precordial lead was identified. The QRS duration was calculated by the algorithms in Muse™, version 9.0 SP6 (General Electric, Boston, USA).

Twenty QRS voltage measurements or combinations were assessed. Some were named after the approach recommended by the first author who proposed the voltage or voltage combination for the diagnosis of LVH, recognizing that LVH was not the objective of this study. The measurements were (1) R wave amplitude in lead AVL (Sokolow) [10], (2) R in lead I (Gubnar) (Gubnar and Ungerleider, 1943), (3) R in lead III, (4) S in Lead V1 (Wilson) [25], (5) S in Lead V2 (Mazoleni) [26], (6) S in lead V3, (7) S in lead V4, (8) R in V4, (9) R in V5 (Wilson) [25], (10) R in V6 (Wilson [25]), (11) S in lead V4 plus deepest S in any precordial leads (Peguero-Lo Presti) [15], (12) S in V1 plus largest R in V5 or V6 [10], (13) S in V3 plus R in AVL (Cornell) [14], (14) S in V2 plus R in V5 or V6 (Romhilt) [27], (15) S in V1 or V2 and R in V5 or V6 (Murphy) [28], (16) S in V1 or V2 plus R in V6 (Grant) [29], (17) R in V5 or V6 (Holt) [30], (18) S in V2 plus R in V4 or V5 (Wolff) [31], and (19) S in V3 plus R in AVL x QRS duration (Molloy/Cornell) [32].

The echocardiographic assessment of the left ventricle included the measurement of left ventricular internal diameter at end diastole (LVID) and end systole (LVIS), left ventricular mass, and LV ejection fraction [33]. LV mass was determined as calculated from 2D echocardiograms and indexed for body surface area (LVMI) [33]. The determination of LV mass was made independent of (without knowledge of) the ECG measurements.

### 2.3. Data Analysis

The data are presented as the mean ± 1 SD. The relation between QRS wave voltages were first assessed using linear regression models. The relation between LV Mass and ECG wave voltages were assessed by linear regression analysis. Nonparametric analysis used the Spearman's rank correlation coefficient model. Multivariate analysis used multiple linear regression models and principal component analysis. A *p* value <0.05 was considered to be statistically significant. The data were analyzed using the statistical software R Studio (Version 1.4.1103) and GraphPad Prism (Version 9.2).

## 3. Results

The baseline characteristics of the 159 individuals in the study show that they were on average 71 years of age and predominantly men (Table 1). The participants had a wide range of cardiovascular diseases. Coronary artery disease was diagnosed by coronary angiogram, CT coronary angiogram, or a nuclear MIBI study.

Focusing on the intercorrelations of QRS voltage showed that there were significant differences in the relationships between the different QRS voltage factors (Table 2). The strongest correlations were, of course, with criteria that had one of its elements, e.g., SV3 and the combination of SV3 plus R in AVL. For the entire study population, S in V3 plus R in AVL correlated highly not only with the product of the R in AVL times QRS duration but also with the S in V3 and SV4 plus deepest S in precordial leads. The strength of the association was less for other leads and combinations, and was not significant for R in leads V4, V5, V6, or lead III. For men, the strength of the relationship between S in V3 plus R in AVL and other voltage indices showed the same pattern (Table 3). The S in V4 plus the deepest S in precordial leads correlates most strongly with, of course, the deepest S in the precordial leads, and S in V3, but not at all with the R wave voltage in leads AVL, I, III, V4, V5, and V6. The magnitude of the S wave in lead V3 correlates most strongly with the S in V4 plus the deepest S in precordial leads and the S in V4 correlated most strongly with the S in V4 plus the deepest S in precordial leads but not at all with the magnitude of the R in V5 or V6.

The pattern of the relations for women is similar to men, except the relationships appear stronger for limb lead criteria but not between the different criteria and any of the following: S V3 plus R in AVL, AVL times QRS duration, SV4 plus deepest S in precordial lead, S in V3 and S in V4 (Table 4).

**Figure 1:**
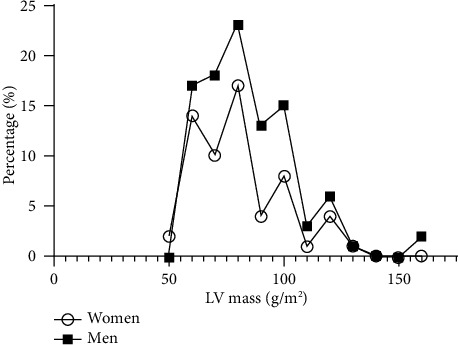
The distribution of LV mass (g/m^2^) for men and women.

The distribution of LV mass showed that the majority of cases did not have left ventricular hypertrophy (Figure 1). Overall, LVH was observed in 23 (14%) individuals in the study group. For men, only 9.1% of cases had an increased LV mass or an LV mass index over 115 g/m^2^. For women, 21.3% of the cases had an increased LV mass or an LV mass index over 95 g/m^2^.

**Figure 2:**
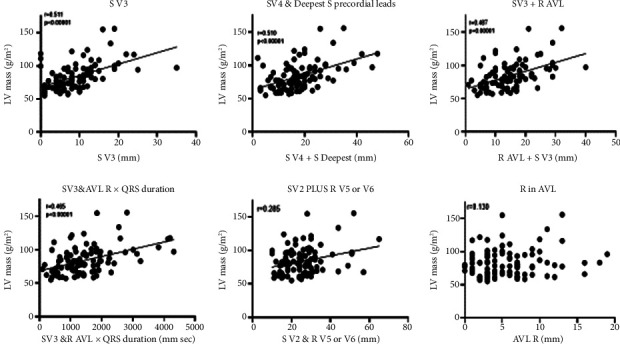
The linear correlation between LV mass and QRS voltage.

The linear regression models of the ECG data showed that the five strongest correlations between QRS voltage and LV mass were S in V4 (*F* = 39.6), deepest S wave in any precordial lead with the S wave in lead V4 (*F* = 33.7), S in V3 (*F* = 28.02), S in V3 plus R in AVL times QRS duration (*F* = 28.04), and S wave in V3 plus R in AVL (*F* = 25.9) (Table 5). For men, the strength of the relationships was S in V3 (*F* = 33.8), sum of the deepest S wave in any precordial lead with the S wave in lead V4 (*F* = 33.7), R in AVL plus S in V3 (*F* = 29.9), and S in V4 (*F* = 29.8). Some of these data were displayed graphically and demonstrated the strong relationship of the S wave amplitude in V3 and LV mass (Figure 2). It also demonstrated that the strength of the correlation diminished when the S in V3 was combined with the R in AVL, which alone did not correlate with LV mass. For women, there was only one voltage criterion that correlated with LV mass and it was the S in V4 (*F* = 7.55).

The relationship between QRS voltages and LV mass were assessed by nonparametric analysis. Similar to the linear regression models, Spearman's correlation models of the ECG data also showed that the sum of the deepest S wave in any precordial lead with the S wave in lead V4 (*r* = 0.408, *p* < 0.0001), S in V4 (*r* = 0.393, *p* < 0.0001), S waves in V3 (*r* = 0.363, *p* < 0.0001), S in V3 plus R in AVL times QRS duration (*r* = 0.339, *p* < 0.0001), S in V3 plus R in AVL (*r* = 0.322, *p* < 0.0001), and S in V2 (*r* = 0.221, *p* < 0.01) were each significantly associated with LV mass. For men, the S in V3 (*r* = 0.545, *p* < 0.0001) and S in V4 (*r* = 0.4622, *p* < 0.001) were significantly associated with LV mass.

**Figure 3:**
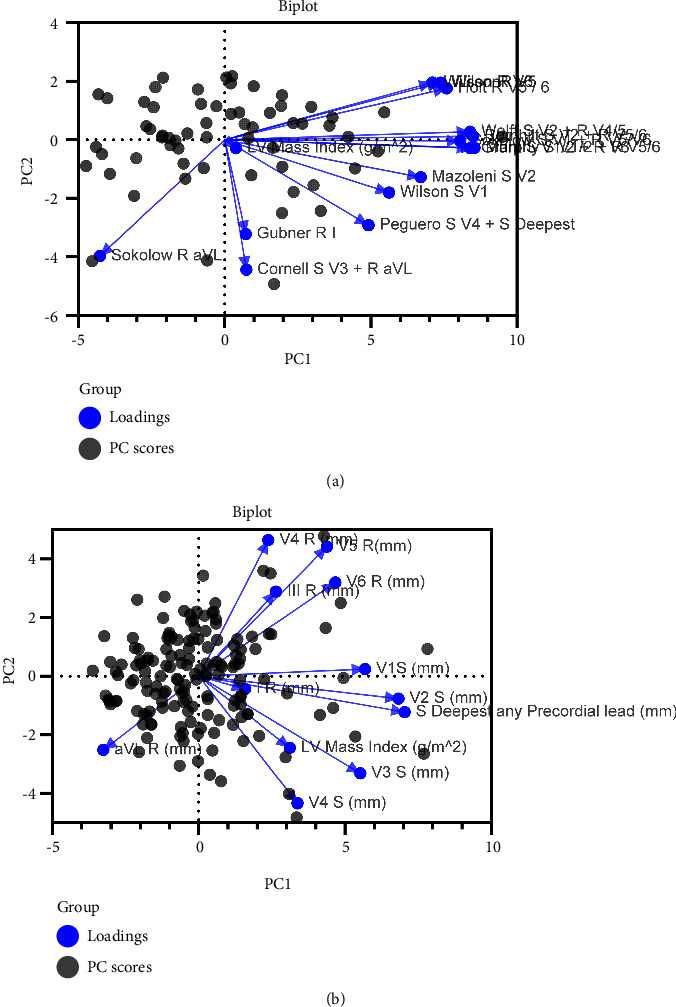
(a) Bioplot from the principle component analysis for men. (b) Bioplot from the principle component analysis for women.

Principal component analysis was used to identify variables that are very similar in nature in order to reduce the number of variables in a large data set. Using the standard ECG criteria, principle component analysis showed that the first two principal components contributed 71.1% of the total variance for men and 54.8% of the total variance for women. Plotting the data in two dimensions shows clusters that are closely related and those that are not (Figures 3(a) and 3(b)). Clearly, the Sokolow criteria of R in AVL and Gubner of R in lead I are very dissimilar from the other criteria.

Multivariate regression was used to determine which of the QRS voltage measurements correlated significantly with LV mass after considering all of the other single measurements of QRS voltage (Table 6). In multivariate analysis, the only significant relationship between QRS voltage and left ventricular mass was for men the S in V3 (*p*=0.04) and for women the S in V4 (*p*=0.016) and R in V6 (*p*=0.04).

## 4. Discussion

This study provides novel information about the QRS voltage interrelationships and the relationship to LV mass in a patient population with a low proportion of LVH. It showed that there were differences between men and women in the relationship of QRS voltage and left ventricular mass. Using multivariate statistical models, it shows that the S wave voltage in V3 (men) and in V4 (women), which are infrequently used criteria for LVH, are the most powerful QRS criteria to estimate LV mass. Furthermore, it showed that criteria using the R wave voltage in AVL are dissimilar from other QRS criteria and is a poor estimator of LV mass.

QRS voltage is proportional to the amount of left ventricular myocardium but it is also influenced by many other factors including LV volume, myocardial fibrosis or myocardial infiltration, and global and regional slowing in conduction velocity in the left ventricle, as well as factors that can affect QRS transmission to the body surface such as lung volumes and anthropometric variables such as body mass index and chest wall thickness [34–38]. These (other) determinants of QRS voltage accounts for the relatively low correlation coefficients between QRS voltage measurements and left ventricular mass which we found and which has been demonstrated by others. Despite these limitations, the ECG is an accepted and frequently recommended method to screen for the presence of left ventricular hypertrophy [24] and to predict cardiovascular outcomes [1–8].

In multivariate analysis, using LV mass as the endpoint and only QRS voltage as covariates we demonstrated the validity of QRS voltage to predict Left ventricular mass. Our study demonstrates that precordial S wave voltages are a more powerful predictor of LV mass than R wave voltages in the limb or precordial leads. The data suggest that an important addition to the assessment of left ventricular mass is the measurement of S waves in leads V3 and V4 rather than the traditional approach of emphasizing the magnitude of the R wave voltage in leads such as AVL and V5 or V6. Our findings are consistent with the conclusion of Peguero et al. who emphasized the value of the deepest S wave in the precordial leads [15]. The data are consistent with the finding that the QRS vector generated by the depolarization of ventricular free wall and myocardium are better represented by the latter part of the QRS complex [39] which results in a greater posteriorly directed vector (precordial S wave) with increased left ventricular mass [15].

The absence of any significant relationship between the R in AVL and LV mass may be explained by the dependency, in part, of the R wave in AVL on the frontal plane QRS axis which in turn is influenced by factors other than left ventricular mass. The absence of a significant relationship between the R in AVL and LV mass accounts for the lack of a relationship between LV mass and other indices that incorporate in their assessment, the R in AVL, such as the R in AVL plus S in V3 and S in V3 plus R in AVL times QRS duration.

It is important to re-emphasize that the purpose of our study was not to develop or examine criteria for LVH. Most studies examining QRS voltage have evaluated the sensitivity and specificity of various ECG criteria for the diagnosis of LVH (for review, see [16]). However, it is interesting to note that other studies have reported that ECG criteria for LVH have different associations with left ventricular mass between men and women. The Cornell voltage-duration product criteria were associated with LVH stronger in women than men whereas presence of ECG-LVH by Sokolow–Lyon voltage criteria was predominantly found in men [40]. It was also not the purpose of our study to examine the ECG in dilated cardiomyopathy which is usually characterized by low electrocardiographic QRS voltages reflecting the loss of cardiomyocytes and the presence of increased cardiac fibrosis [41]. Our study did not address low QRS voltage but by inference, it may be in the low voltage group.

An advantage of our approach is that it relies on a single measurement of QRS complex rather than incorporating other factors such as ST or T waves, *p* wave amplitude, QRS duration, or QRS-T wave axis discordance [42]. Interestingly some investigators contend that ST-T wave changes can be a stronger indicator of LVH than QRS voltage [43]. However, those investigators used R wave voltage in AVL which we have identified is a poor indicator of LV mass.

Our data are different from those of Verdecchia et al. who studied untreated patients with hypertension (mean age 49 years, 46% women) without LVH on ECG and no history of cardiovascular disease [22]. They reported that the sum of the S wave in V3 plus the R wave in AVL showed the closest association with LV mass (*r* = 0.36) and progressively weaker correlations were shown with the R wave in lead I (*r* = 0.25), the S wave in lead III (*r* = 0.22), and the S wave in lead V3 (*r* = 0.19) [22]. The differences may relate to the differences in the study populations as our study was on average an older age, more men and had a variety of cardiovascular diseases as opposed to only hypertension. It is important, however, to recognize that QRS voltage is a significant predictor of subsequent cardiovascular events not only in patients with hypertension but also in patients with other kinds of cardiovascular diseases including coronary artery disease and heart failure [5, 7, 8].

### 4.1. Limitations of the Study

It is important to emphasize that this was a retrospective study which has the limitations inherent in this kind of study. However, the kinds of ECG data collected are unlikely to have been different in a prospective study. Second, the sample size was relatively small but it required an in-depth measurement of QRS complex which cannot readily be done in large samples. Third, the assessment of left ventricular mass was based on the echocardiogram and not a MRI assessment of the heart which is more accurate. Although it was not possible for us to systematically collect information on MRI in these patients, it is likely that very few patients had cardiac MRI because of the nature of the patient population which was one without previous myocardial infarction, left or right bundle branch block, pre-excitation, or electronic pacemaker. However, in an analysis of 26 longitudinal echocardiographic studies and 5 MRI studies investigating LV mass and LV hypertrophy, either modality was a reliable cardiovascular risk predictor of death or major cardiovascular outcomes [44]. Fourth, the study population was selected as a consecutive set of patients attending the clinic so that by chance there were more men than women. Fifth, information on the presence of chronic obstructive lung disease or chronic kidney disease, which may affect LV mass or QRS voltage, was not available. Sixth, the study focused on a reflection of LV mass in the ECG. It adjusted for the effect of body size on QRS by the standard approach using body surface area. Interestingly, it has been suggested that LVH determined by ECG and echocardiography predict mortality independently of each other and of other cardiovascular risk factors, implying that ECG and echocardiographic assessment of LV mass carry different prognostic information [45]. Thus, ECG assessment of LV mass may have special importance underscoring the necessity for its use in cardiovascular risk assessment [45].

## 5. Conclusion

Univariate and multivariate analysis showed a strong correlation between some but not all QRS voltages and left ventricular mass. The magnitude of the S wave in lead V3 or V4 was identified in multivariate analysis to be the strongest QRS factor correlating with left ventricular mass. In contrast, the R wave in limb leads, including AVL, did not correlate with LV mass. The addition of the R wave in AVL to precordial QRS voltage measurements reduces the strength of the association of precordial QRS voltages with LV mass. Considering that QRS voltage is a significant predictor of subsequent cardiovascular events in patients with different kinds of cardiovascular diseases including coronary artery disease and heart failure [5, 7, 8], using QRS voltage to assess prognosis should consider refocusing on the S wave in precordial leads, in patients even in the absence of ECG evidence of LVH [46].

## Figures and Tables

**Table 1:** Characteristics of the study population.

Age (years)	70.8 ± 15.4
Sex (M/F)	99/60
Hypertension	50.3%
CAD	14.5%
Arrhythmias	19.5%
Valvular heart disease	17.0%
Heart failure or cardiomyopathy	5.7%
Aortic aneurysm	2.5%
Men LV mass (g/m^2^)	84.4 ± 20.0
EF (%)	59.9 + 6.7
LVID (mm)	47.6 ± 5.7
LVIS (mm)	31.2 ± 5.6
Women LV mass (g/m^2^)	79.4 ± 18.8
EF %	60.6 ± 5.4
LVID (mm)	44.5 ± 4.7
LVIS (mm)	27.6 ± 4.6

**Table 2:** The correlation between QRS voltage in some leads.

	S V3 + R aVL	AVL R × QRS duration	S V4 + S deepest precordium	V3S	V4S
aVL R (Sokolow)	0.574^*∗∗∗∗*^	0.485^*∗∗∗∗*^	0.095	0.0124	0.223^*∗∗*^
I R (Gubnar)	0.433^*∗∗∗∗*^	0.317^*∗∗∗∗*^	0.096	−0.029	0.025
III R	−0.175	−0.082	0.105	0.0503	−0.139
V1S (Wilson)	0.363^*∗∗∗∗*^	0.352^*∗∗∗∗*^	0.548^*∗∗∗∗*^	0.336	0.096
V2 S (Mazoleni)	0.518^*∗∗∗∗*^	0.547^*∗∗∗∗*^	0.819^*∗∗∗∗*^	0.739^*∗∗∗*^	0.363^*∗∗∗∗*^
V3 S	0.826^*∗∗∗∗*^	0.809^*∗∗∗∗*^	0.867^*∗∗∗∗*^	1	0.721^*∗∗∗∗*^
V4 S	0.717^*∗∗∗∗*^	0.740^*∗∗∗∗*^	0.781^*∗∗∗∗*^	0.721^*∗∗∗∗*^	1
V4 R	−0.209^*∗*^	−0.234^*∗∗*^	−0.057	−0.129	−0.287^*∗∗∗*^
V5 R (Wilson)	−0.04	−0.072	0.139	0.039	−0.123
V6 R (Wilson)	0.089	0.073	0.233^*∗∗∗*^	0.128	0.037
S deepest any precordial lead	0.597^*∗∗∗∗*^	0.611^*∗∗∗∗*^	0.896^*∗∗∗∗*^	0.744^*∗∗∗∗*^	0.421
S V4 + S deepest (Peguero)	0.763^*∗∗∗∗*^	0.784^*∗∗∗∗*^	1	0.866^*∗∗∗∗*^	0.781^*∗∗∗∗*^
S V1 + R V5/V6 (Sokolow)	0.2114^*∗∗*^	0.196^*∗*^	0.426^*∗∗∗∗*^	0.239^*∗*^	0.04
S V3 + R aVL (Cornell)	1	0.936^*∗∗∗∗*^	0.763^*∗∗∗∗*^	0.826^*∗∗∗∗*^	0.7167^*∗∗∗∗*^
S V2 + R V5/6 (Romhilt)	0.341^*∗∗∗∗*^	0.350^*∗∗∗∗*^	0.632^*∗∗∗∗*^	0.511^*∗∗∗∗*^	0.213^*∗∗∗*^
S V1/2 + R V5/6 (Murphy)	0.339^*∗∗∗∗*^	0.341^*∗∗∗∗*^	0.630^*∗∗∗∗*^	0.465^*∗∗∗∗*^	0.185^*∗*^
S V1/2 + R V6 (Grant)	0.387^*∗∗∗∗*^	0.389^*∗∗∗∗*^	0.665^*∗∗∗∗*^	0.504^*∗∗∗∗*^	0.228^*∗∗∗*^
R V5/6 (Holt)	0.043	0.029	0.220^*∗∗∗*^	0.104	−0.012
S V2 + R V4/5 (Wolff)	0.243^*∗∗∗*^	0.245^*∗∗∗*^	0.554^*∗∗∗∗*^	0.446	0.114
AVL R × QRS duration (Molloy Cornell)	0.936^*∗∗∗∗*^	1	0.784^*∗∗∗∗*^	0.804^*∗∗∗∗*^	0.740^*∗∗∗∗*^

Correlation coefficient (*r*) is listed as 1 when the factor is correlated with itself. ^*∗*^*p* < 0.05, ^*∗∗*^*p* < 0.01, ^*∗∗∗*^*p* < 0.001, and ^*∗∗∗∗*^*p* < 0.0001.

**Table 3:** The correlation between QRS voltage in some leads for men.

	S V3 + R aVL	AVL R × QRS duration	S V4 + deepest S in precordium	V3S	V4S
aVL R (Sokolow)	0.581^*∗∗∗∗*^	0.507^*∗∗∗∗*^	0.096	0.039	0.204∗
I R (Gubnar)	0.480^*∗∗∗∗*^	0.373^*∗∗∗*^	0.112	0.036	0.014
III R	−0.127	−0.052	0.175	0.09	−0.091
V1S (Wilson)	0.488^*∗∗∗∗*^	0.455^*∗∗∗∗*^	0.608^*∗∗∗∗*^	0.463^*∗∗∗∗*^	0.193
V2 S (Mazoleni)	0.568^*∗∗∗∗*^	0.571^*∗∗∗∗*^	0.829^*∗∗∗∗*^	0.784^*∗∗∗∗*^	0.395^*∗∗∗∗*^
V3 S	0.836^*∗∗∗∗*^	0.810^*∗∗∗∗*^	0.892^*∗∗∗∗*^	1	0.722^*∗∗∗∗*^
V4 S	0.700^*∗∗∗∗*^	0.728^*∗∗∗∗*^	0.793^*∗∗∗∗*^	0.722^*∗∗∗∗*^	1
V4 R	−0.328	−0.3568	−0.155	−0.207	−0.41
V5 R (Wilson)	−0.063	−0.141	0.084	0.013	−0.179
V6 R (Wilson)	0.126	0.054	0.220^*∗*^	0.133	0.024
S deepest any precordial lead	0.637^*∗∗∗∗*^	0.633^*∗∗∗∗*^	0.896^*∗∗∗∗*^	0.787^*∗∗∗∗*^	0.439^*∗∗∗∗*^
S V4 + S deepest (Peguero)	0.779^*∗∗∗∗*^	0.79^*∗∗∗∗*^	1	0.892^*∗∗∗∗*^	0.793^*∗∗∗∗*^
S V1 + R V5/V6 (Sokolow)	0.266^*∗∗*^	0.205^*∗*^	0.423^*∗∗∗∗*^	0.290^*∗∗*^	0.061
S V3 + R aVL (Cornell)	1	0.937^*∗∗∗∗*^	0.779^*∗∗∗∗*^	0.836^*∗∗∗∗*^	0.701^*∗∗∗∗*^
S V2 + R V5/6 (Romhilt)	0.381^*∗∗∗*^	0.343^*∗∗∗*^	0.630^*∗∗∗∗*^	0.549^*∗∗∗∗*^	0.219^*∗*^
S V1/2 + R V5/6 (Murphy)	0.370^*∗∗∗*^	0.331^*∗∗∗*^	0.620^*∗∗∗∗*^	0.496^*∗∗∗∗*^	0.186
S V1/2 + R V6 (Grant)	0.440^*∗∗∗∗*^	0.400^*∗∗∗∗*^	0.669^*∗∗∗∗*^	0.544^*∗∗∗∗*^	0.243^*∗*^
R V5/6 (Holt)	0.037	−0.028	0.176	0.088	−0.049
S V2 + R V4/5 (Wolff)	0.24	0.2	0.522^*∗∗∗∗*^	0.455^*∗∗∗∗*^	0.084
AVL R × QRS duration (Molloy Cornell)	0.939^*∗∗∗∗*^	1	0.790^*∗∗∗∗*^	0.810^*∗∗∗∗*^	0.728^*∗∗∗∗*^

Correlation coefficient (*r*) is listed as 1 when the factor is correlated with itself. ^*∗*^*p* < 0.05, ^*∗∗*^*p* < 0.01, ^*∗∗∗*^*p* < 0.001, and ^*∗∗∗∗*^*p* < 0.0001.

**Table 4:** The correlation between QRS voltage in some leads for women.

	S V3 + R aVL (Cornell)	AVL Rx QRS duration (Molloy Cornell)	S V4 + S deepest precordium (Peguero)	V3S R	V4S R
aVL R (Sokolow)	0.480^*∗∗∗∗*^	0.299	−0.049	−0.195	0.156
I R (Gubnar)	0.258^*∗*^	0.073	−0.025	−0.2813	−0.040
III R	−0.219	−0.051	0.059	0.046	−0.180
V1S (Wilson)	0.064	0.095	0.44	0.035	−0.173
V2 S (Mazoleni)	0.319^*∗*^	0.440^*∗∗∗*^	0.789^*∗∗∗∗*^	0.589^*∗∗∗∗*^	0.201
V3 S	0.767^*∗∗∗∗*^	0.794^*∗∗∗∗*^	0.762^*∗∗∗∗*^	1	0.674^*∗∗∗∗*^
V4 S	0.705^*∗∗∗∗*^	0.716^*∗∗∗∗*^	0.690^*∗∗∗∗*^	0.674^*∗∗∗∗*^	1
V4 R	−0.189	−0.253	−0.012	−0.121	−0.233
V5 R (Wilson)	−0.155	−0.082	0.173	0.0003	−0.136
V6 R (Wilson)	−0.108	0.023	0.211	0.056	−0.0145
S deepest any precordial lead	0.420^*∗∗∗*^	0.499^*∗∗∗∗*^	0.893^*∗∗∗∗*^	0.588^*∗∗∗∗*^	0.290^*∗*^
S V4 + S deepest (Peguero)	0.649^*∗∗∗∗*^	0.714^*∗∗∗∗*^	1	0.762^*∗∗∗∗*^	0.690^*∗∗∗∗*^
S V1 + R V5/V6 (Sokolow)	−0.045	0.045	0.384^*∗∗*^	0.0435	−0.1379
S V3 + R aVL (Cornell)	1	0.906^*∗∗∗∗*^	0.649^*∗∗∗∗*^	0.767^*∗∗∗∗*^	0.705^*∗∗∗∗*^
S V2 + R V5/6 (Romhilt)	0.1111	0.247	0.592^*∗∗∗∗*^	0.361^*∗∗*^	0.072
S V1/2 + R V5/6 (Murphy)	0.121	0.233	0.604^*∗∗∗∗*^	0.316^*∗*^	0.041
S V1/2 + R V6 (Grant)	0.143	0.27∗	0.624^*∗∗∗∗*^	0.348^*∗∗*^	0.08293
R V5/6 (Holt)	−0.117	−0.008	0.2278	0.038	−0.07
S V2 + R V4/5 (Wolff)	0.082	0.193	0.567^*∗∗∗∗*^	0.347^*∗∗*^	0.024
AVL R x QRS duration (Molloy Cornell)	0.906^*∗∗∗∗*^	1	0.714^*∗∗∗∗*^	0.794^*∗∗∗∗*^	0.717^*∗∗∗∗*^

Correlation coefficient (*r*) is listed as 1 when the factor is correlated with itself. ^*∗*^*p* < 0.05, ^*∗∗*^*p* < 0.01, ^*∗∗∗*^*p* < 0.001, and ^*∗∗∗∗*^*p* < 0.0001.

**Table 5:** Shows the correlation between various leads and lead combinations with left ventricular mass.

	Men and women	Men	Women
aVL R	0.102	0.130	−0.012
I R	0.047	0.101	−0.094
III R	−0.018	−0.045	0.055
V1S	0.162^*∗*^	0.325^*∗∗∗*^	−0.1371
V2 S	0.254^*∗∗*^	0.324^*∗∗∗*^	0.076
V3 S	0.389^*∗∗∗∗*^	0.511^*∗∗∗∗*^	0.068
V4 S	0.449^*∗∗∗*^	0.487^*∗∗∗∗*^	0.337^*∗∗*^
V4 R	−0.102	−0.114	−0.191
V5 R	0.025	0.028	−0.032
V6 R	0.161	0.193	0.074
S deepest any precordial lead	0.318^*∗∗∗∗*^	0.396^*∗∗∗∗*^	0.116
S V4 + S deepest in any precordial lead	0.440^*∗∗∗∗*^	0.510^*∗∗∗∗*^	0.246
S V1 + R V5/V6	0.160^*∗*^	0.253^*∗*^	−0.053
S V3 + R aVL	0.376^*∗∗∗∗*^	0.487^*∗∗∗∗*^	0.053
S V2 + R V5/6	0.224^*∗*^	0.285^*∗∗*^	0.061
S V1/2 + R V5/6	0.231^*∗∗*^	0.299^*∗∗*^	0.05
S V1/2 + R V6	0.267^*∗∗∗*^	0.342^*∗∗∗*^	0.081
R V5/6	0.114	0.132	0.029
S V2 + R V4 or V5	0.165^*∗*^	0.209^*∗*^	0.019
S V3 + R aVL x QRS duration	0.389^*∗∗∗∗*^	0.465^*∗∗∗∗*^	0.148

Correlation coefficient (*r*) is listed as 1 when the factor is correlated with itself. ^*∗*^*p* < 0.05, ^*∗∗*^*p* < 0.01, ^*∗∗∗*^*p* < 0.001, and ^*∗∗∗∗*^*p* < 0.0001.

**Table 6:** Multiple linear regression analysis between QRS voltage and left ventricular mass.

QRS voltage in lead	Women	*P* value	Men	*P* value
	Intercept		Intercept	
aVL R (mm)	0.1311	0.8758	−0.09960	0.9236
I R (mm)	−0.3464	0.6889	−0.1353	0.9013
III R (mm)	−0.1269	0.7894	−0.3412	0.5955
V1S (mm)	−0.1163	0.8431	0.6577	0.3690
V2 S (mm)	−1.079	0.1471	−1.383	0.1196
V3 S (mm)	0.2341	0.6901	1.523	0.0365^*∗*^
V4 S (mm)	1.480	0.0158^*∗*^	0.9038	0.1992
V4 R (mm)	0.06761	0.8550	0.4381	0.3517
V5 R (mm)	−0.7544	0.2716	−1.027	0.1849
V6 R (mm)	1.200	0.0397^*∗*^	1.216	0.0605
S deepest any precordial lead (mm)	1.568	0.0871	0.8171	0.4546

^*∗*^ *p* < 0.05.

## Data Availability

The data used in this study are not available because of patient rights (confidentiality).
